# Reasons why life on Earth rarely makes fluorine-containing compounds and their implications for the search for life beyond Earth

**DOI:** 10.1038/s41598-024-66265-w

**Published:** 2024-07-06

**Authors:** Janusz J. Petkowski, Sara Seager, William Bains

**Affiliations:** 1 Department of Earth, Atmospheric and Planetary Sciences, Massachusetts Institute of Technology, 77 Massachusetts Avenue, Cambridge, MA 02139 USA; 2 Faculty of Environmental Engineering, Wroclaw University of Science and Technology, 50-370 Wroclaw, Poland; 3 JJ Scientific, Warsaw, Mazowieckie Poland; 4 Department of Physics, Massachusetts Institute of Technology, 77 Massachusetts Avenue, Cambridge, MA 02139 USA; 5 Department of Aeronautics and Astronautics, Massachusetts Institute of Technology, 77 Massachusetts Avenue, Cambridge, MA 02139 USA; 6 School of Physics & Astronomy, Cardiff University, 4 The Parade, Cardiff, CF24 3AA UK; 7 Rufus Scientific, Melbourn, Royston, Herts, UK

**Keywords:** Astrobiology, Astrobiology

## Abstract

Life on Earth is known to rarely make fluorinated carbon compounds, as compared to other halocarbons. We quantify this rarity, based on our exhaustive natural products database curated from available literature. We build on explanations for the scarcity of fluorine chemistry in life on Earth, namely that the exclusion of the C–F bond stems from the unique physico-chemical properties of fluorine, predominantly its extreme electronegativity and strong hydration shell. We further show that the C–F bond is very hard to synthesize and when it is made by life its potential biological functions can be readily provided by alternative functional groups that are much less costly to incorporate into existing biochemistry. As a result, the overall evolutionary cost-to-benefit balance of incorporation of the C–F bond into the chemical repertoire of life is not favorable. We argue that the limitations of organofluorine chemistry are likely universal in that they do not exclusively apply to specifics of Earth’s biochemistry. C–F bonds, therefore, will be rare in life beyond Earth no matter its chemical makeup.

## Introduction

Life does not make representatives of all the types of chemicals that could be constructed from the non-metallic elements that makeup biochemistry. There are some unexpected lacunae in the coverage of stable chemical space by the chemistry of life. Exploring why life fails to fully use chemistry in these ‘‘gaps’’ is as important as explaining why life does exploit a specific atom, bond, or molecule class found in biochemistry^1–3^. Such explanations illuminate the chemical nature and evolution of life on Earth, and address whether profoundly different chemistry could be the basis of life on other worlds^4^. There are several areas of chemical space that appear to be avoided by life. The recently published quantified examples and mechanistic explanations for such “gaps” in Earth-life’s biochemistry include the relative absence of chemical bonds between nitrogen and sulfur atoms (N–S bonds) in biochemistry^5,6^, the exclusion of trivalent phosphorus chemistry from the chemical repertoire of life^4,7^ or the absence of organosilicon functional groups^8^. We hypothesize that the existence of such avoided areas of chemical space is a specific example of a more general phenomenon: the chemistry that life can use is limited not only by obvious environmental constraints such as the availability of elements or stability of molecules in water but also by a range of more subtle constraints on how a self-consistent biochemistry can be assembled^4,5^. In this paper we explore another such biochemical “gap” in the coverage of stable chemical space, the exclusion of organofluorine (fluorocarbon) compounds, particularly the exclusion of the C–F bond.

The rarity of organofluorine compounds in Earth's biochemistry is well known and there are many excellent reviews that give a thorough overview of the chemistry and biochemistry of fluorine-containing natural products and the biology of species that make them^9–13^. The physcio-chemical properties of organofluorines and the C–F bond in particular is also a well-studied and important topic in both biology and chemistry (e.g.^14,15^). However, the rarity of fluorine in Earth’s biochemistry is not broadly known and is not yet recognized in planetary science or astrobiology.

We first review established knowledge on the chemistry of the C–F bond and the rarity of natural fluorocarbons in Earth’s biochemistry and provide new, quantitative information on the occurrence of C–F bond-containing natural products (sect. “The rarity of fluorine in Earth’s life”). We follow our quantitative analysis with a discussion of barriers limiting the biosynthesis of the C–F bond, first reviewing established concepts and next proposing new rationale behind life’s exclusion of F from its biochemistry (Sect. “Potential interdictors of fluorine as a major component of biochemistry”). We present the implications of the exclusion of the C–F bond from Earth’s biochemistry for the search for life beyond Earth (Sect. “Implications for the search for life beyond Earth”).

## Methods: custom natural products database

To quantify the extent to which life on Earth produces F-containing compounds we use our database of natural product chemicals curated over the last decade. Our database is described in^5^. We created and curated our database by an extensive literature search and by searching available online natural product repositories. For description of the subset of our natural product database that contains volatile molecules see^16^.

Our natural products database has been rigorously screened to contain only compounds that are a result of natural biochemical processes of life. We specifically exclude products of human chemistry, such as synthetic derivatives of natural products or synthetic chemicals that interfere with natural processes, such as drugs or pesticides. The database also identifies biological sources for every entry (i.e., data on the species from which the natural product was isolated).

To ensure the completeness of our collection of natural fluorine-containing molecules we have also performed an exhaustive manual literature search. Hundreds of new natural products are being discovered every year in both marine and terrestrial organisms and it is likely that, although they are rare on Earth, more F-containing natural products await discovery. For context, in 1968 there were only 24 naturally occurring organohalogens known, currently organohalogen natural products number more than 5000, as they continue to be discovered in all regions of the world^17,18^.

We note that the natural product chemistry is increasingly difficult to study due to ubiquitous pollution of the natural environment with human industrial chemicals. Human industrial pollution especially affects the proper identification and enumeration of rare functional groups and chemical motifs that life on Earth generally avoids, i.e. the “gaps” in chemical space^5^. Each novel detection of a rare chemical motif has to be scrutinized not only with respect to the proper structure determination of the isolated chemical (as exemplified by the incorrect claims of biological production of 3,5-di-tert-butyl-4-fluorophenylpropionic acid, a compound containing a fluorine atom bonded to an aromatic carbon in the benzene ring^19–21^) but also by confirmation, without any doubt, that the putative natural product is a genuine biochemical and not an industrial contaminant metabolized and accumulated in the cells of a living organism.

The human industrial pollution affects the discovery of new natural products containing fluorine in particular, as F is a ubiquitous element in the chemistry used by human pharmaceutical and agricultural industry. The extent of human industrial pollution is so vast that even organisms perpetually living at the bottom of Earth’s deepest ocean trenches, such as the Mariana trench, are exposed to human industrial chemicals^22^. Such ubiquitous contamination puts serious doubt on any reports of new fluorine-containing biochemicals isolated from the natural sources. As the human impact on the biosphere and environment increases, the search, discovery and confirmation of rare, novel and unique biochemicals evolved by life on Earth will become increasingly difficult. Eventually the confirmation that a newly discovered natural products containing a rare functional groups or chemical motifs will be extremely difficult, if not utterly impossible without simultaneous elucidation of biosynthetic pathways for each of the newly discovered compounds. Examples of such controversial natural products that could be the result of industrial contamination include fluoro-uracils (**27**–**31**)^23^, fluoromethyl ester of 2-amino-2-phenylacetic acid (**23**) or 2’-fluoro-2’-deoxyadenosine (**26**)^24^. Compounds (**23**) and (**26**) isolated from maggots of oriental latrine fly *Chrysomya megacephala* resemble clinically significant fluorinated nucleoside drugs and could be contaminants excreted by humans and then accumulated by the insect larvae^25^. Therefore, the natural product status of compounds (**23**, **26**–**31**) awaits confirmation.

## The rarity of fluorine in Earth’s life

Fluorine is known to be rarely used by Earth’s life. In this Section we review and quantify the rare occurrence of organofluorine natural products (Sect. “Rare occurrence of fluorocarbons in Earth life’s natural products in the context of non-fluorine natural halocarbons”) and review (with new examples) the extent to which life on Earth uses inorganic fluorine (Sect. “Use of inorganic fluorine by life on Earth”).

### Rare occurrence of fluorocarbons in Earth life’s natural products in the context of non-fluorine natural halocarbons

**Figure 1:**
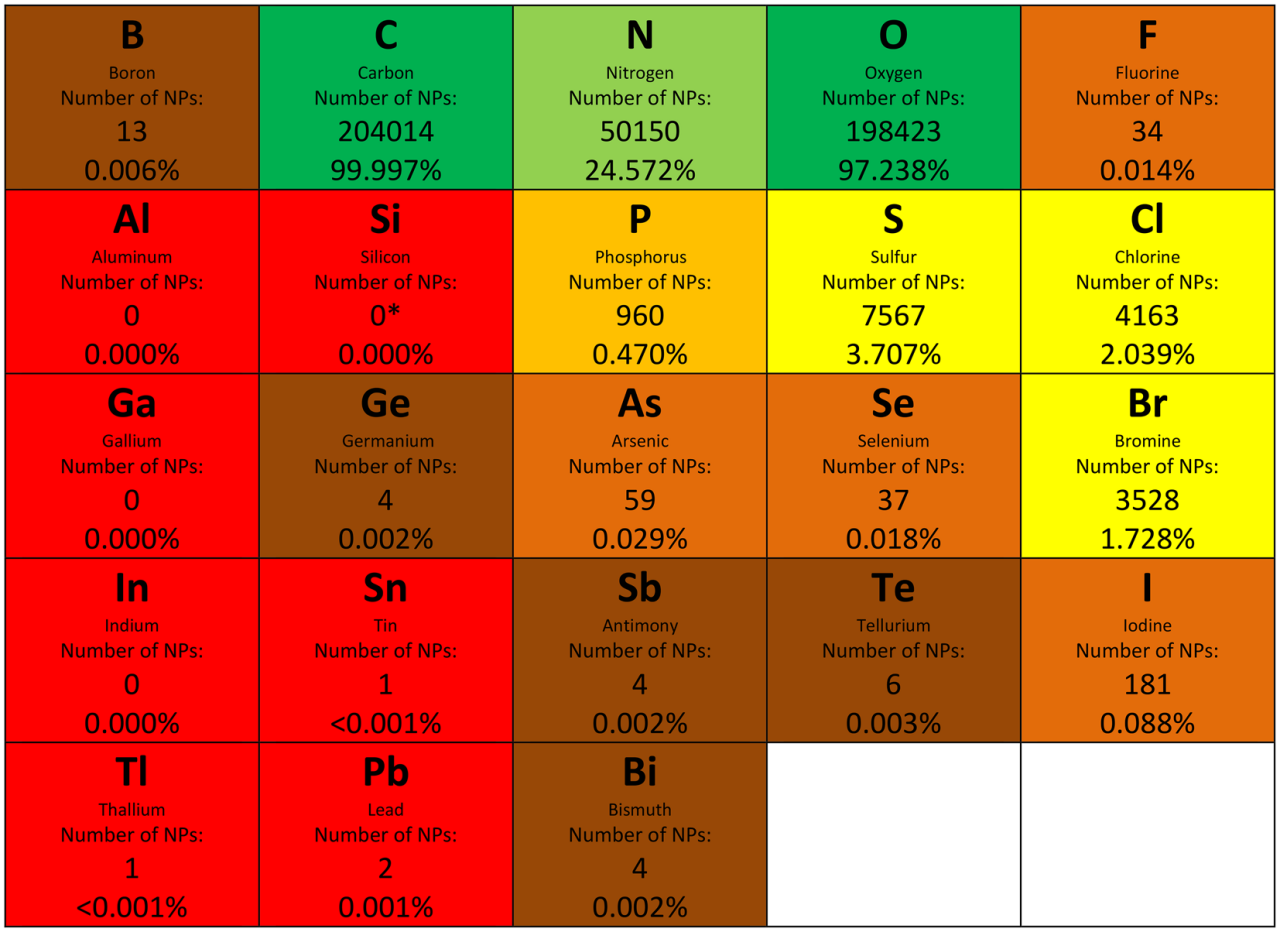
Elemental abundances amongst natural products (NPs). The number of natural products in our database^5^ containing a given element and the percentage number are shown. The halogen atoms Cl- and Br-containing compounds are nearly as common as S–containing compounds amongst natural products. In contrary to Cl, Br and even I halogens, F-containing natural compounds are severely underrepresented in the chemical repertoire of life on Earth. The figure compilation shows only elements that can form covalent bonds that are stable in water. The compilation excludes transition metals from the analysis. (*) No molecules containing Si bonded to any atom other than oxygen are known to be made by life, although silica and silicic acid are used extensively by life on Earth^8^.

Fluorocarbons are extremely rare in life’s products, even though other halocarbons are quite common. Halocarbons (organohalogens) are compounds with carbon atoms covalently bonded to halogen atoms: fluorine (F), chlorine (Cl), bromine (Br), or iodine (I). Compounds within the halocarbon group include many pharmaceutical drugs as well as the human-made volatile chlorofluorocarbons. Life produces thousands of halocarbons. Most are Cl-containing compounds, with Br-containing compounds a close second, and F-containing compounds are nearly excluded. To illustrate the rarity of F-containing compounds, we have enumerated compounds with each element in the P-block of the periodic table from our database described in Sect. “Methods: custom natural products database” (Fig. 1). Out of about 200,000 documented unique natural products, over 2% contain Cl. This number is quadruple that of phosphorus. About 4% of natural products contain a halogen atom, as high as natural products containing sulfur. In total, about 5000 natural products contain halogens^18^, a fraction of which are volatile^16,26^. While halocarbons are not part of the core metabolism of life as sulfur is, halocarbons are still an abundant and a very important part of Earth life’s biochemistry.

We emphasize that the halogen–containing compounds produced by life are nearly all halocarbons. The only other classes of halogenated compounds produced by life are S–halogens (e.g. S–Cl and S–Br bond, found in proteins as an intermediary in synthesizing the N–S bonds^5^) and N–halogens (N–Br, N–Cl). Both S–halogens and N–halogens are quite reactive and therefore rare. Notable examples of N–halogens are N–chlorotaurine^27^, N–bromotaurine^28^, pseudoceratonic acid^29^, and potentially a few others^30,31^ (see Supplementary Appendix B for the list of structures of known natural N–halogenated compounds). O-halogens are only produced by life as reactive HOX species (HOCl, HOBr, HOI), e.g., during the immune response, as a chemical weapon against invading bacteria^32^. All of the small number of fluorine-containing compounds made by life are fluorocarbons, even though compounds with F linked to N or S are stable^26^. (We note that compounds containing F–O bonds are implausible biological products, being extremely reactive and powerful oxidizing and fluorinating agents, and in some cases explosively unstable.)

Nearly all of the known biogenic fluorocarbons are fluorinated carboxylic acids (Appendix A). These include, e.g., 13 different variants of fluorinated fatty acids (**1**–**13**) produced by the tropical plant *Dichapetalum toxicarium* or fluoroacetic acid (**16**) produced by many different species of bacteria and plants. Although terrestrial plants provide most of the known natural organofluorines, *Streptomyces* bacteria also produce several such compounds. Notable examples include toxic amino acid fluorothreonine (**18**)^33^ or antibiotic nucleocidin (**21**)^34^.

A series of 5-fluorouracils (**27**–**31**) from the sponge *Phakellia fusca* have also been isolated^23^, but their true origin as naturally produced chemicals remains to be confirmed, and some authors question their natural product status^11,35^.

For a full overview and list of chemical structures of known fluorine-containing molecules produced by life on Earth for the Supplementary Appendix A. For thorough previous reviews of organofluorine compounds produced by life on Earth see^9–13^.

**Figure 2:**
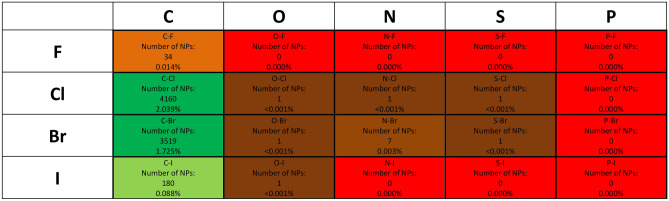
Abundance of chemical bonds containing halogens (F, Cl, Br, I) and biogenic elements (C, O, N, S, P) among natural products (NPs). The figure depicts the number of natural products in our database^5^ containing chemical bonds with halogen atoms and the percentage number of all natural products containing these chemical bonds. The numbers show that not all bonds between halogen atoms and the five biogenic elements are equally frequent amongst natural products, some bonds are more common (C–Cl, C–Br, C–I) than others (C–F, N–Br) and some are very rare in Earth’s biochemistry.

To close this subsection, we re-emphasize that the number of natural products containing S–F, N–F, P–F and O–F bond is strictly zero (Fig. 2).

### Use of inorganic fluorine by life on Earth

The scarcity of fluorine in terrestrial biochemistry (Sect. “Rare occurrence of fluorocarbons in Earth life’s natural products in the context of non-fluorine natural halocarbons”) refers to the rarity of compounds where fluorine is covalently linked to carbon (i.e. in organic molecules), and the absence in biochemistry of compounds where F is linked to nitrogen, sulfur or oxygen. Several authors have postulated that Earth life’s near avoidance of F is due to F’s overall rarity in the Universe and more specifically the low concentration of fluorine in sea water^36,37^. However the apparent low bioavailability of fluorine cannot be the explanation for the rarity of fluorine in organic biochemicals, as fluorine is used extensively in other chemistries. Inorganic fluoride (e.g. as F^-^ ions) is known to be widely present in the biosphere^38^. Terrestrial plants, e.g. from the genus *Camellia*, which includes the commercial tea plant *Camellia sinensis*, can selectively concentrate inorganic fluoride from relatively low concentrations in the soil^9^. Fluoride is also a minor component of many animal skeletal structures, including inter alia human teeth. A truly extreme example of fluorine’s use in a skeleton comes from the marine sponge *Halichondria moorei*. Although the concentration of fluoride in seawater is only ~ 1.3 ppm, 11.5% of the dry body weight of the sponge *Halichondria moorei* constitutes fluorine. *Halichondria moorei* expends significant amount of energy to capture and concentrate F^-^ ions from the surrounding sea water to convert them into potassium fluorosilicate (**25**) (K₂SiF₆) in its skeleton^39^. We note that a closely related sponge *Hymeniacidon perleve* that lives in the same habitat as *Halichondria moorei* does not contain detectable fluorine^39^. The sponge habitat is free of fluorine except for the small amount dissolved in seawater as fluoride^39^. The example of the *Halichondria moorei* illustrates that low availability of fluorine in the environment does not pose an obstacle for life as long as there is an evolutionary advantage to utilizing that element. Indeed, environmental scarcity is seldom a barrier to an element’s use. Life widely uses elements that are much rarer than fluorine. Note that iodide, which is much less abundant than fluoride in the ocean has been identified in ~ 180 natural products, compared to fluorine’s 34 (Fig. 1). Selenium, for instance, is used universally in metabolism, in archaea, bacteria, and eukaryotes^3^, despite Se being 10000 times rarer in the crust^40^ and 30000 times rarer in seawater than fluorine^41^. Even essential elements like zinc and iron are less abundant in seawater than fluorine. The barrier to fluorine’s use must therefore lie not in acquiring fluoride but in converting fluoride to organofluorine compounds.

By contrast to Earth’s use of inorganic F only a few organofluorine compounds are made by life (as mentioned above in Sect.  “Rare occurrence of fluorocarbons in Earth life’s natural products in the context of non-fluorine natural halocarbons"), and with the exception of potassium fluorosilicate (**25**) no compounds are made where fluorine is linked to atoms other than carbon, despite the stability of some of these compounds^26^. Synthesis of organofluorines occurs only in a very restricted group of organisms. We next discuss the potential barriers limiting the biosynthesis of organofluorines.

## Potential interdictors of fluorine as a major component of biochemistry

**Figure 3:**
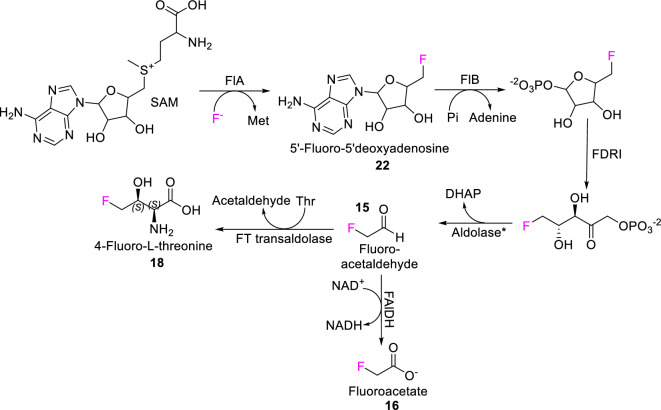
Biosynthesis of C–F bonds. The first native fluorinase that has been characterized is a nucleophilic halogenase, flA from *Streptomyces cattleya*. The fluorinase flA, isolated from *S. cattleya* in 2002^72^, catalyses C–F bond formation from an inorganic fluoride ion. FlA fluorinase mediates a reaction between *S*-adenosyl-L-methionine (SAM) and a fluoride ion to yield 5’-fluorodeoxyadenosine (**22**) and L-methionine, the first step in the biosynthesis of fluoroacetate (**16**) and 4-fluorothreonine (**18**). *SAM* *S*-adenosyl-L-methionine, *Met* L-methionine, *Pi* phosphate, *DHAP* dihydroxyacetone phosphate, NADH/NAD^+^: reduced and oxidized variants of nicotinamide adenine dinucleotide, FlA: Fluorinase, *FlB* 5′-fluoro-5′-deoxyadenosine phosphorylase, *FDRI* 5-fluoro-5-deoxyribose-1-phosphate isomerase, *FAlDH* fluoroacetaldehyde dehydrogenase, *FT transaldolase* fluorothreonine transaldolase. *putative aldolase responsible for the formation of fluoroacetalaldehyde (**15**). Figure modified and adapted from^37^.

The degree to which any innovation is adopted by life depends on a balance between the evolutionary cost and benefit of evolving a new adaptation. Thus, the evolutionary cost-to-benefit balance changes with the ecological setting of the organism. Such a cost-to-benefit relationship also applies to incorporating novel biochemical solutions, such as the biosynthesis of the organofluorine compounds, into metabolism (Fig. 3, Table 1). We consider that such evolutionary barriers fall into two classes: the cost of making organofluorine compounds (Sect. “Costs of making organofluorine compounds”), and the paucity of unique benefits that organofluorine compounds provide (Sect. “Potential selective value of organofluorines”).

**Table 1:** Apparent cost-to-benefit balance of including organofluorine in Earth’s biochemistry.

Incorporation of organofluorine chemistry into biochemistry: costs, benefits and existing evolutionary solutions		
Costs of incorporating C–F chemistry	Potential evolutionary benefit of C–F chemistry	Existing non C–F evolutionary solutions
Extreme electronegativity makes biosynthesis of the C–F bond a challenge	To provide a hydrophobic group to a molecule, to increase the molecule’s lipophilicity and ability to penetrate membranes	Many aliphatic (e.g. alkanes) and aromatic (e.g. phenyl) hydrophobic groups can increase lipophilicity of compounds
The chemical resistance of the C–F bond makes the C–F bond very stable once formed	To block a site of metabolic attack	There are many ways to block the site of metabolic attack that do not require organofluorine chemistry
The F^−^ ion is very strongly hydrated in solution; any fluorine-utilizing enzymatic machinery must expend energy to remove fluorine’s hydration shell	As a weak H-bond acceptor	Readily available functional groups without F but instead containing S, O, N atoms or π electron clouds can act as weak H-bond acceptors
Repurposing of other enzymes (i.e. defluorinases, haloperoxidases) to make C–F bonds is not possible	To enable phase separation or phase transfer	The wide spread use of intrinsically disordered proteins or lipid rafts
	As part of a ‘warhead’ in an irreversible inhibitor	There are many ways to irreversibly inhibit enzyme catalytic activity with non-fluorine chemistry

### Costs of making organofluorine compounds

The evolutionary costs of making organofluorine compounds arise from the difference between fluorine and other halogens. Fluorine is not ‘just another halogen’, in at least several respects that make the adoption of organofluorine chemistry very costly for life (Table 2). However, the costs of adoption of organofluorine chemistry for an organism does not mean that evolving a fluorinase (an enzyme that catalyzes the formation of the C–F bond) is an extremely improbable, ‘one time’ event, in the same sense that the evolution of oxygenic photosynthesis was a complex, one-time event. Fluorinases appear to have evolved several times, even though the catalytic mechanism of the known fluorinases appears to be the same, the result of convergent evolution.

**Table 2:** Selected physical properties of halogen atoms. Sources:^9,15^.

Element (X)	Pauling electronegativity scale	Hydratation energy, X^-^ (kcal/mol)	van der Waals’ radius (Å)	Standard ox./red. potential E^0^ (2X^-^ = X_2_ + 2e) (V)	Dipole moment (C-X) (debye)	C-X bond length (Å)		Bond energy (kcal/mole)	
						C–X	H–X	C–X	H–X
F	4.0	117	1.47	−2.87	1.82	1.35	0.92	107	147.5
Cl	3.0	84	1.80	−1.36	1.94	1.78	1.27	66.5	102.7
Br	2.8	78	1.95	−1.07	1.97	1.93	1.41	54.0	87.3
I	2.5	68	2.15	−0.54	1.64	2.14	1.61	45.5	71.4
H	2.2	–	1.20	–	0	1.09	–	98.8	–

#### Fluorine is more electronegative than any other element

The unique physical and chemical properties of fluoride, dominated by its extreme electronegativity, have made the evolution of C–F bond biosynthesis a significant challenge for life. We review fluorine’s extreme electronegativity and describe implications for the costs of utilization of F in biochemistry.

First, the enzymatic machinery of haloperoxidases that generates other halocarbons is based around peroxidases, i.e. enzymes that handle moieties with an electrode potential of around 1.8 V. Such enzymes are widespread in biology, but they cannot be repurposed to fluoridate compounds. Thus, fluorinating enzymes can be repurposed to handle the less electronegavtive chloride ion, but the reverse is mechanistically impossible (a problem that we discuss in more detail in Sect. “Fluorination and defluorination of organic molecules follow two completely unrelated catalytic mechanisms”). Fluorinating enzymes must therefore be evolved de novo. Life on Earth has evolved an impressive collection of halogenating enzymes capable of introducing chlorine, bromine, and iodine into a wide range of biochemicals^42^. The enzymatic halogenation proceeds through the oxidative formation of reactive halogen species, halogen radicals or cations. Fluorine doesn’t participate in biosynthesis as cationic species unlike other halogens and is not readily oxidized by haloperoxidases and therefore cannot be readily incorporated into biochemicals^43^.

Haloperoxides use H_2_O_2_ as an oxidizer to oxidize Cl^−^, Br^−^ and I^−^ to their respective hypohalites (HOX, where X is Cl, Br or I). Hypohalites are then used to introduce halogens into biochemicals. Many natural products containing Cl, Br and I are biosynthesized in this way and much of the biochemistry of the Cl, Br and I involves the oxidation of halide ions (X^−^) to halonium ions (X⁺) or halide radicals (X·)^43–46^. Peroxidases in turn are universal in biochemistry, where handling reactive oxygen species has been a prerequisite of life probably from its origin. Thus, life is pre-adapted to develop the enzymatic machinery for Cl, Br and I biochemistry. However haloperoxidases cannot oxidize F^−^ because the oxidation potential of H_2_O_2_ (−1.8 eV) is above that of fluoride (F^−^ =  −2.87 eV), although they can oxidize the other halogens whose oxidation potential (Cl^−^  =  −1.36 eV; Br^−^  =  −1.07 eV; I^−^  =  −0.54 eV) is below that of H_2_O_2_^47^. The prevalence of I in natural products, unlike F, is likely because iodide is readily oxidized by haloperoxidases, and life’s enzymatic machinery can generate iodonium ion (I⁺) through the same mechanism as Cl^+^ and Br^+^.

Secondly, very highly oxidizing species (fluorine included) are handled poorly by biochemistry in general. A well-studied example of this challenge, outside of the fluorine chemistry, is a formation of ferryl iron (Fe(IV)), which is a rare side-product of oxygen chemistry in haemoglobin in mammals. The standard redox potential of FeO_3_^2–^/Fe^3+^ is 2.2 V. Ferryl ion is highly toxic, and specific protein machinery has evolved to remove it rapidly if it is formed^48,49^. Thus fluorine-handling enzyme machinery would need to protect the rest of the cell from fluorine’s detrimental reactivity as well as perform the intended relevant chemistry.

#### The fluoride ion is very strongly hydrated in solution

Even if enzymatic machinery could be evolved that handled oxidized fluoride species, the hydration of the fluoride ion raises a thermodynamic barrier to organofluorine synthesis. Fluorine has a small ionic radius and as a consequence is very strongly hydrated in solution. Fluoride is the most strongly hydrated halide, much stronger than Cl^–^, Br^–^, or OH^–^. While it is well known that F^–^ has a strong hydration shell, we have to emphasize the result is that any aqueous fluorine chemistry must expend substantial energy dehydrating the ion before it can be bonded to another atom. Fluoride’s very tight shell of hydration must be removed to allow the formation of a C–F bond. Fluorine has the highest heat of hydration (∼120 kcal mol^−1^) of all the halogens (Table 2); therefore, to achieve nucleophilic catalysis in an aqueous environment^9^, an efficient desolvation strategy for F^−^ has to be evolved first.

The additional energy expenditure adds to the overall costs of utilization of organofluorines in biochemistry. In some cases reaction of fluoride in water can be enabled by incorporation of surfactants, where the surfactant partially de-solvates the fluoride at the water:surfactant interface^50^, analogous to desolvation of fluoride at the active site of fluorinases. However, this limitation is a reason why almost all human synthetic fluorine chemistry is carried out in non-aqueous solvents, where the fluoride ion is much more reactive.

#### The carbon–fluorine bond is very chemically resistant

The carbon–fluorine bond is very resistant to chemical alteration (sometimes described as it being a very ‘strong’ bond) and the C–F bond ‘strength’ increases with additional fluorine substituents on an aliphatic carbon^14,15,51^. The chemical resistance of the C–F bond justifies many of the applications of fluorocarbons in high temperature and/or corrosive environments^14^. For example, the C–F bond in natural product fluoroacetate (**16**) is stable to boiling concentrated sulfuric acid^52^ and the complete liberation of fluoride from fluoroacetate, i.e. breaking of the C–F bond, requires very harsh conditions e.g. sodium fusion at 500 °C degrees or refluxing in 30% NaOH^53,54^. In contrast to the stability of the C–F bond, the other carbon-halogen bonds are quite labile (Table 2). Moreover, because of the electronegativity difference between carbon and fluorine (2.5 vs 4.0) (Table 2), C–F bonds are highly polar, this polarity further contributes to the overall strength of the C–F bonds. Electron withdrawal by the fluorine atoms often generally strengthens the neighboring skeletal bonds in the molecule, as it e.g. happens in fluorocarbons. The C–C bond in hexafluoroethane (C_2_F_6_) is 7 kcal/mol stronger than the corresponding C–C bond in ethane (C_2_H_6_)^14^.

In order to form a C–F bond C and F have to pass through a transition state where both C and F are only partially bonded to other atoms. As F is monovalent, this is a very high energy state, making the C–F bond very stable once formed. We address the specific challenges of fluorination and defluorination in the next section.

#### Fluorination and defluorination of organic molecules follow two completely unrelated catalytic mechanisms

In many metabolic pathways, the synthetic and degradative mechanisms are related, so a degradase enzyme can sometimes adapt to be ‘reversed’ to act as a synthesase. A well-known example of such an evolutionary adaptation is the ‘reversal’ of the classical tricarboxylic acid cycle (TCA) to form an anabolic pathway^55^. However, such ‘reversal’, i.e. adaptation of defluorinases (enzymes that cleave C–F bond and remove fluorine from organofluorine compounds) to make C–F bonds cannot apply to organofluorine chemistry, as we describe next.

The chemical stability of C–F bond poses a challenge for biodegradation of organofluorines. Direct cleavage of the C–F bond in monofluorinated compounds, like fluoroacetate, proceeds readily with defluorinases, while di- and trifluoroacetate are intact^56^. Defluorinases, have been identified in many different organisms from diverse branches of the tree of life^57^, from bacteria^58^ to animals^59,60^,yeast^61^ and fungi^62^. They can remove fluorine from a very wide variety of substrates, both aliphatic and aromatic organofluorines, in both aerobic as well as anaerobic conditions^63–69^. Enzymes that handle other substrates can also be bioengineered into defluorinases with relatively few modifications (e.g.^70^). The defluorinase enzymes are especially widespread in animals that inhabit areas colonized by the fluoroacetate-producing plants, where defluorination is part of the detoxification mechanism of the toxic organofluorine natural products produced by plants^71^.

Defluorination produces a hydrated fluoride ion and uses the energy of hydration of that ion to drive the reaction. The defluorination mechanism typically involves nucleophilic attack on the carbon to which fluorine is attached, a very efficient mechanism as the carbon–fluorine bond is highly polarized. Similar catalytic mechanisms are widespread in other enzyme classes (e.g. esterases, peptidases). Evolution of such mechanisms is possible, as illustrated by an enzyme aconitase. This key enzyme in the tricarboxylic acid cycle (TCA) is poisoned by fluoroacetate. Instead of converting citrate to isocitrate, aconitase catalyzes the removal of fluoride by nucleophilic attack by OH^−^, to form hydroxyaconitate, which binds very tightly to the enzyme and inhibits it. Aconitase is therefore an accidental defluorinase, albeit one whose products poison it. (See below for further discussion of the toxic effects of fluorine compounds.)

Neither aspect of the defluorination reaction can readily be reversed to add a fluorine atom to a carbon. The chemistry required for the formation of the C–F bond is entirely different from the mechanism of its breakage. A well-studied example of such a C–F bond forming enzyme is fluorinase flA from a bacterium *Streptomyces cattleya*. FlA facilitates the nucleophilic attack of a desolvated fluoride ion on *S*-adenosyl-L-methionine (SAM) to create a C−F bond yielding 5′-fluorodeoxyadenosine (5′-FDA) (**22**), which is further processed into fluoroacetate and 4- fluorothreonine (Fig. 3). The example of the flA fluorinase shows that the biological formation of new C–F bonds requires novel evolutionary approaches that are distinct from the rest of the halogen biochemistry. Incorporating F into biochemical repertoire of life requires new unique catalytic solutions developed specifically for fluorine. Fluorinase flA from *Streptomyces cattleya*^72^, and its close orthologs in related bacterial species (e.g.^73^), are as of yet the only known native enzymes that generate the C–F bond.

The biosynthetic challenge of making C–F bonds is further illustrated by the fact that fluorinase SAM enzyme activity can be repurposed for chlorination, bromination or iodination with just few amino acid mutations in its active site, while the change of activity of chlorinases, brominases or iodidinases to fluorinase is not possible^42^. For example, SalL is an enzyme from the marine bacterium *Salinispora tropica* that is closely related to fluorinase flA. SalL can catalyze chlorination, bromination, and iodination, but has lost its capacity for fluorination^74^.

With such apparently limited ways to make C–F bond in nature are there any other means by which life makes C–F bonds that await discovery, or is the reaction between SAM and the F^−^ ion the only way that life on Earth could make C–F bonds? The metabolic origin of the fluoroacetate in all of the ~ 30 plant species known to produce it remains unknown, as is the identity of the C–F forming enzyme and the involved biosynthetic pathway. It is therefore unknown if plants use the same biochemical mechanism as bacteria to make fluoroacetate or if they have evolved some other unique chemistry to make the C–F bond.

Another mystery is the biosynthesis of the antibiotic nucleocidin (**21**)^75^. The structure of nucleocidin is sufficiently distinct that the bacteria producing it must contain a fluorination enzyme that differs from the fluorinase involved in fluoroacetate biosynthesis^75–81^, even if both fluoroacetate and nucleocidin contain the same C(sp^3^)−F bond. Even greater mystery is the potential formation of fluorouracils by marine sponges^23^ (if those are indeed genuine products of sponge metabolism). The enzymatic strategy for fluorine incorporation into aromatic heterocycles requires entirely novel catalytic mechanism. Nevertheless, if confirmed, this opens the possibility that more fluorinases await discovery, maybe even enzymes capable of the formation of the sp^2^-bound fluorine, C(sp^2^)−F bond.

We conclude that the evolution of organofluorine biochemistry requires a range of adaptations that are unique to fluorine chemistry, and are not simple modifications or repurposing of other, existing halogen or oxygen chemistry. New, specialized enzymatic machinery is needed specifically to make organofluorine compounds. This is not an insuperable problem for life but implies that strong selective reason for evolving such a unique enzyme machinery must exist if its evolution is to be favored. The metabolic cost of maintaining the ability to fluorinate organic molecules is supported by the observation that some bacteria that cannot themselves make organofluorine compounds nevertheless have halogenase enzymes homologous to fluorinases, and hence presumably evolved from them. The ancestors of bacteria containing these fluorinase-like halogenases were able to make organofluorine compounds, but the selective benefit of doing so was not sufficient to retain fluorinase function. Insufficient evolutionary benefit led to repurposing the C–F bond forming enzymes to make other organohalogen compounds instead.

### Potential selective value of organofluorines

We cannot know what benefit fluorine could have conferred in an imaginary world where the organofluorines are widely biosynthesized. Relying on scientific imagination to predict what biology can and cannot do is fraught with problems, as illustrated by numerous examples of biology that have been declared biologically impossible only to have subsequently been discovered to exist, such as tolerance of extremes of radiation^82^, growth at temperatures above 100 °C^83^, even survival in Earth orbit (ISS)^84^. However, in the case of fluorine chemistry we can use human industry, especially the pharmaceutical industry, as a proxy for evolutionary innovation to probe potential useful biological functions for organofluorine compounds.

The rarity of the fluorine-containing biochemicals contrasts heavily with a large number of organofluorine molecules used in diverse branches of human industry, which illustrates the diverse functional value of F chemistry^85^. Since the discovery of fluorocortisone in 1953^86,87^ an entire industry has explored the use of fluorine in drugs and other bioactive agents^88,89^. Organofluorine compounds have been widely used in pharmacology and medicine^90–93^. More than 20% of drugs in clinical trials contain a fluorine atom^93^ and 45% of all small molecule drugs approved by the U.S. Food and Drug Administration in 2018–2019 contained fluorine^94^. Fluorinated chemicals are also widely used in agriculture (16% of all launched pesticides contain fluorine^95^; this number goes to 52% agrochemicals approved by the FDA 2010–2017^96^). Organofluorines are also widely used in material science^97–101^.

The prevalence of fluorine compounds in human industry illustrates that the potential biological value of organofluorine chemistry has been well explored. The widespread and diverse pharmacology of the C–F bond supports the idea that organofluorine compounds could have functional and biologically useful properties and that life could use fluorine to a powerful effect. If that is indeed true, then why has life not evolved the capability to exploit the potential of the C–F bond? To answer this question, we consider what role fluorine plays in the molecules that the combined pharmaceutical and agrochemical industries have discovered. We show that the role fluorine plays in human chemical industries can be summarized in five categories (a–e) and that the potential selective value of organofluorines in all of those potential roles of fluorine chemistry can be substituted by other existing biochemical solutions that do not use F.

**(a) To provide a hydrophobic group to a molecule, to increase its lipophilicity and ability to penetrate membranes.** The C–F bond has a ‘polar hydrophobic’ character that allows molecules containing C–F functional groups to stably dissolve in both polar and hydrophobic solvents. The ‘polar hydrophobicity’ of organofluorines exist due to electron withdrawing power of the C–F bond that reduces the polarizability and increases the hydrophobicity of molecules^15^. This increased hydrophobicity of C–F bond allows for greater lipophilicity and membrane penetration of organolfuorines as compared to their non-F analogs. Combined with the chemical stability of the C–F bond this feature makes organofluorines an attractive target for drug design. However, biochemistry can achieve additional lipophilicity and membrane penetration using exclusively carbon chemistry in a wide variety of ways, for example with hydrophobic side chains of amino acids, both aliphatic and aromatic, or various hydrocarbons and other hydrophobic membrane penetrating small molecule natural products. It is also not clear that an ability of a water-soluble compound to penetrate membranes is an advantage, as cells use the distinction between hydrophilic and lipophilic metabolites as a mechanism for keeping the contents of cellular compartments distinct and *not* mixing with each other, as mentioned below.

**b) To block a site of metabolic attack**, such as in gemcytabine (reviewed in^102^) and possibly in natural product nucleocidin (**21**). Again, a wide range of substitutions exist in biology to avoid metabolic degradation. Among the many examples we might cite the incorporation of thiophosphorate deoxynucleotides in bacterial DNA to block phage nucleases^103^, and the use of D-amino acids as long-lived neurotransmitters in mammals^104^. Biology also can generally modulate metabolism and stability of compounds by compartmentalization, such as the compartmentalization of oxygen-sensitive nitrogenase in cyanobacterial heterocysts^105^, reactive nitrogen-sulfur bonds inside protein molecules^6^, and proteases inside lysosomes and autophagosomes where they degrade target proteins and not the whole cell^106^. This last example illustrates the different constraints on the pharmaceutical chemistry and the evolved cell. The cell can partition molecules into compartments to modulate their breakdown as it is e.g. in the case of partitioning proteins to target them for degradation by proteases. The pharmaceutical chemistry must aim to design molecules that have to act systemically throughout the cell (and the entire body). This constraint is specific to pharmaceutical chemistry and is a reason why pharmaceutical chemistry prefers stabilization of drug molecules by introducing chemically resistant C–F bonds.

**c) As a weak H-bond acceptor**. A common use of a fluorine atom in drug or agrochemical design is as a hydrogen bond acceptor^15,107,108^. Alternative chemistries that are readily available for life can provide for weaker hydrogen bond acceptors than O or N atoms. Such weak hydrogen bond acceptors alternatives as S, Se or amide nitrogen, are not favored in medicinal chemistry because of undesirable metabolic properties or chemical reactivity of these groups. Evolution can also change the hydrogen bond donor to a weaker variant to attain the same effect, for example replacing O–H with S–H or even acetylenic C–H or other C–H groups (e.g.^109^). Pharmaceutical chemistry cannot engineer the protein to which the drug has to bind, so modification of proteins by introducing weaker H-bonds is a path open to evolution but not to technology, which is why human industry prefers the use of fluorine as a weak H-bond acceptor.

**d) To enable phase separation or phase transfer**. Fluorine atom has low polarizability, which results in weak cohesive forces between fluorocarbon molecules, this in turn translates to exceptional volatility of this class of molecules^14^. For example, perfluorohexane (C_6_F_14_) boils at 57 °C, 12 °C below hexane (C_6_H_14_) despite having a molecular weight roughly four times larger^14^. It also means that fluorocarbons often form unique phases that are immiscible with both aqueous and non-aqueous solvents. This feature can promote the self-assembly of complex organized molecular structures e.g., formed by some perfluorocarbons (reviewed in^36^). In biomedicine these properties of fluorine are readily exploited, e.g. in the case of the C_8_F_18_ as a ‘splint’ in retinal surgery^110^. However, in biochemistry liquid:liquid phase changes are readily achieved in cells using intrinsically disordered proteins^111,112^, and lipid rafting^113^. The use of disordered proteins and lipid rafts allows for multiple phases within the aqueous compartment of the cell without the need for a difficult C–F bonds or any chemistry other than the canonical biochemistry.

**Figure 4:**
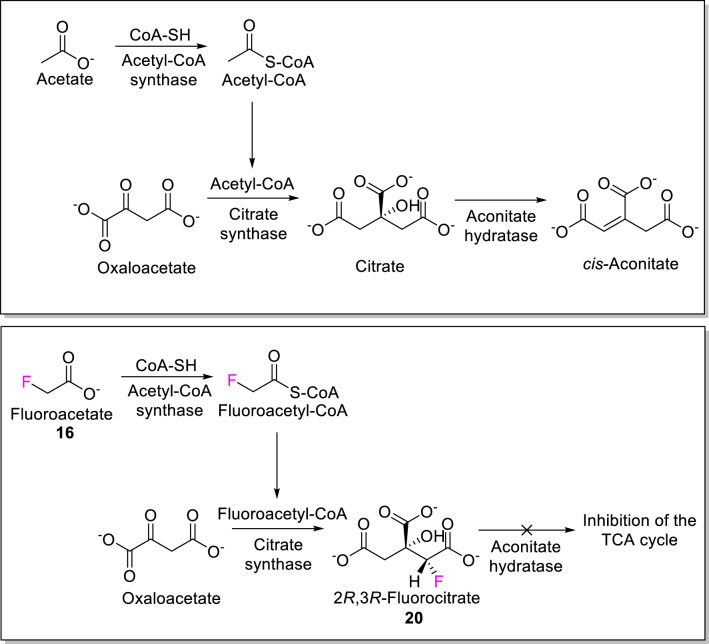
Production of toxic fluorocitrate (**20**) inhibitor of the tricarboxylic acid cycle (TCA). Upper panel: TCA cycle generating *cis*-aconitate through the action of the enzyme aconitate hydratase; lower panel: inhibition of aconitate hydratase as a result of fluorocitrate production. Figure adapted and modified from:^13^.

**e) As part of a ‘warhead’ in an irreversible inhibitor.** All the natural molecules containing F produced by life on Earth are toxic. Their universal toxicity is plausibly the main reason behind their biological function, as they are used as specialized “chemical weapons” against predation. The best-known example is fluoroacetate’s blockade of the tricarboxylic acid cycle by conversion of fluoroacetate (**16**) to fluoroacetyl-CoA and subsequently to (2*R*,3*R*)-fluorocitrate (**20**) (Fig. 4). Fluorocitrate is a strong inhibitor of the enzyme aconitase (which converts it to hydroxyaconitate which strongly inhibits the enzyme as noted above), as well as an inhibitor of mitochondrial transmembrane citrate transport^13^. No other analogue of acetate could play this role.

The mode of action of other toxic fluorine-containing natural products, when known, often appears to involve toxic fluoroacetate as well. For example, the toxicity of fluorocarboxylic fatty acids (**1**–**13**) depends on the number of carbon atoms in the fatty acid chain. If the total number of carbon atoms in the fluorinated fatty acid chain is even the compound is toxic, if it is odd, it is safe. The toxic fluorinated fatty acids are degraded via the β-oxidation of fatty acids pathway which degrades fatty acids in two-carbon steps. Even-numbered fatty acids end up with fluoroacetate, which is toxic, whereas odd-numbered fatty acids end up with 3-fluoropropanoic acid, which as it cannot be used in the tricarboxylic acid cycle is non-toxic to animals^114^. Therefore, the universal toxicity of F-containing natural products is dependent on the very specific mode of action, tailored to specific metabolic pathways and biochemistry, just a reflection of their specific intended biological function based on structural mimics of core biochemicals (e.g. fluoroacetate (**16**)). The benefit of organofluorine compounds and the stability of the C–F bond might, therefore, be strongest in organisms that wish to deter animal predation (in which compounds need to be delivered through a digestive system). Such organisms must survive in harsh environments where growth is slow, and toxins have to be stored for a long time against occasional predation. This conclusion is supported by finding organofluorine compounds e.g. in desert plants^115^.

In many instances, life has readily adapted to the toxic effect of fluorine and toxic natural organofluorines. Examples of such adaptations are quite common and include: targeted defluorination of toxic organofluorines with dedicated defluorinase enzymes^59,60,116^ or utilization of fluoride transporters^117,118^. Several insects that feed on fluorine-rich plants accumulate and metabolize fluorine in their tissues, possibly using the accumulated F to synthesize their own unique F-containing natural toxins^9^.

Fluorine compounds, and especially fluorine compounds of phosphorus, are indubitably powerful irreversible enzyme inhibitors. However other halogen compounds can also provide irreversible inhibition. Examples include methyl chloride and bromide, both of which are widely produced by algae and believed to act as broad-spectrum biocidal agents; methyl bromide is also used by human pest controllers for the same purpose. Even the aconitase enzyme mentioned above, specifically targeted by fluoroacetate to interfere with the TCA cycle, can be inhibited through other means, for example via succination of critical cysteine residues in its active site^119^, to yield the same effect. In addition, other chemistries can potently block oxidative phosphorylation, such as natural products oligomycin^120^ and rotenone^121^, which are as potent inhibitors of oxidative phosphorylation as fluoroacetate, and contain no halogen atoms.

We conclude that extensive human exploration of the biochemical potential of organofluorine chemistry suggests that the benefits that fluorine confers on a compound can be readily duplicated with other, less challenging chemistry by living organisms.

## Implications for the search for life beyond Earth

In the search for life beyond Earth, fluorine has largely been discussed in terms of technosignature gases (that is, gases produced from technology such as chlorofluorocarbons e.g.,^26,122^). The previous sections on the costs and barriers to implementation of organofluorine in biochemistry that stem from the physico-chemical properties of fluorine, its extreme electronegativity, strong hydration shell and other characteristics have consequences for life beyond Earth. The limitations of organofluorines are likely universal and do not exclusively apply to specifics of Earth’s biochemistry. If life elsewhere develops novel biochemistry to exploit oxidized species, of which fluorine is one example, by far the most likely oxidized species would be oxidized forms of oxygen. Oxygen is the third most abundant element in the universe, it is present in the most likely solvents for life (H_2_O, H_2_SO_4_, CO_2_)^123^, and handling O is a key component for fixing CO_2_, the most abundant source of inorganic carbon.

Thus, the barriers to introducing fluorine for an alien biochemistry are likely the same as for Earth life. Exceptions could only occur when: (a) fluorine is much more abundant than it is on Earth^36^, which seems unlikely as the Hypatia catalogue does not show any stars with a huge excess of fluorine over oxygen that are known to host planets^124,125^; (b) oxidation of the planetary environment is much higher than on Earth, which also seems unlikely, as such alien environment would have to be more oxidizing than Earth’s surface, which is exposed to 21% O_2_ in the atmosphere; or (c) the stability of the C–F bond is of substantial benefit.

Taking advantage of the exceptional stability of the C–F bond is the only plausible option, and could refer e.g. to high temperature environments such as ocean worlds with near-critical water oceans. However, such a high-temperature ocean environment raises issues of sufficient stability for the other chemistry that life would need to use in that environment.

A speculative evolutionary application of the C–F bond stability in an alien biochemistry could include its role as a specialized mimic of a C–H bond. Fluorine is the closest atom in size to hydrogen atom (Table 2). Due to its relatively small size and a short and very strong bond to carbon fluorine can substitute hydrogen atoms in virtually every type of organic molecule, making such substitutions a unique feature of fluorine chemistry^14^. The ability of fluorine to “cap” carbon atoms in organic molecules, and therefore structurally mimic and substitute hydrogen, could provide a unique advantage for life in planetary environments where water (surface of Mars) or hydrogen atoms in general (clouds of Venus) remain scarce. No other atom or a functional group used to “cap” carbon atoms of molecules comes closer to the size of hydrogen.

As an aside, some authors speculated on the possibility of liquid hydrogen fluoride (HF) as a potential alternative solvent for life on planetary bodies that are substantially colder than Earth^36^. The idea is that life that uses liquid HF instead of water as a solvent for its biochemistry would also use C–F bond in its biomolecules to much greater extent than Earth life does. However, it is hard to imagine an environment where substantial amounts of liquid HF would be present on a rocky planet for two reasons^123^. Firstly, volcanoes outgas far more water than HF. Therefore, for the surface liquid HF reservoirs to form, as opposed to a water ocean containing dissolved HF, volcanic gases would have to contain negligible oxygen, i.e., no water, while at the same time having at least some hydrogen. We do not know of any suggestion of how a rocky planetary crust could form that is sufficiently oxygen-depleted while containing some hydrogen. Secondly, HF reacts with silica so any putative surface reservoir of liquid HF would react with crustal rocks to form fluorides and water. These two limitations suggest that the only potential environment where liquid HF could potentially exist as a planetary solvent is on hypothetical carbide planets^123^. On such planets carbon is more abundant than oxygen and therefore oxygen is outgassed exclusively as CO_2_. Carbide planets have been postulated^126^, but whether they exist is unknown.

## Conclusions

There are several classes of chemicals that life appears to avoid^4–8^. In this paper we have continued the exploration of such biochemical “gaps” in the coverage of stable chemical space by explaining the exclusion of organofluorine compounds from the chemical repertoire of life. In contrast to the previous studies on why life fails to use chemistry in these ‘‘biochemical gaps’’, which could be explained by specific reactivity of the excluded chemistry with specific components of Earth’s biochemistry^4,5^, the probable reasons for the exclusion of the C–F bond are more complex. The C–F bond is very hard to make and when made its potential biological functions can be readily provided by alternative functional groups that are much less costly to incorporate into existing biochemistry. As a result, the overall evolutionary cost-to-benefit balance of incorporation of the C–F bond into the chemical repertoire of life is not favorable.

### Supplementary Information

Supplementary Information.

## Data Availability

The data is provided in the supplementary information (SI) file. The authors are also willing to provide the original datasets on request, please contact Dr. Janusz J. Petkowski (jjpetkow@mit.edu).
