# Supplementary material for: Reasons why life on Earth rarely makes fluorine-containing compounds and their implications for the search for life beyond Earth
Source: Sci Rep. 2024 Jul 6;14:15575. doi: 10.1038/s41598-024-66265-w (PMC11227584; doi:10.1038/s41598-024-66265-w)
Supplement: Supplementary file 1 — Supplementary Information. [file 41598_2024_66265_MOESM1_ESM.docx]

Supplementary Information

Reasons Why Life on Earth Rarely Makes Fluorine-containing Compounds and Their Implications for the Search for Life Beyond Earth

Janusz J. Petkowski ^1,2,3^*, Sara Seager ^1,4,5^ and William Bains ^1,6,7^

^1^ Department of Earth, Atmospheric and Planetary Sciences, Massachusetts Institute of Technology, 77 Massachusetts. Avenue., Cambridge, MA 02139, USA

^2^ Faculty of Environmental Engineering, Wroclaw University of Science and Technology, 50-370 Wroclaw, Poland

^3^ JJ Scientific, Mazowieckie, Warsaw, Poland

^4^ Department of Physics, Massachusetts Institute of Technology, 77 Massachusetts. Avenue., Cambridge, MA 02139, USA

^5^ Department of Aeronautics and Astronautics, Massachusetts Institute of Technology, 77 Massachusetts. Avenue., Cambridge, MA 02139, USA

^6^ School of Physics & Astronomy, Cardiff University, 4 The Parade, Cardiff CF24 3AA, UK

^7^ Rufus Scientific, Melbourn, Royston, Herts, UK

******* Correspondence: [jjpetkow@mit.edu](mailto:jjpetkow@mit.edu)

**Supplementary Appendix A:** List of chemical structures of known fluorine-containing molecules produced by life on Earth.

| **Compound no.** | **Comments** | **Reference** |
| --- | --- | --- |
| **1** |  | ^1^ |
| **2** |  | ^1^ |
| **3** |  | ^1^ |
| **4** |  | ^2^ |
| **5** |  | ^3^ |
| **6** |  | ^2,4^ |
| **7** |  | ^5^ |
| **8** |  | ^2^ |
| **9** |  | ^2^ |
| **10** |  | ^2^ |
| **11** |  | ^2,3^ |
| **12** |  | ^3^ |
| **13** |  | ^2^ |
| **14** | Produced by many species, including as an F^-^ ion in defluorinase reaction. | ^6,7^ |
| **15** | Precursor of fluoroacetate (**16**) and 4-fluorothreonine (**18**) in *Streptomyces cattleya*. | ^8^ |
| **16** | Produced by many plant and *Streptomyces* species. | ^9–14^ |
| **17** | Its status as a genuine natural product remains to be confirmed ^15^. | ^16^ |
| **18** |  | ^8,9,13,17–19^ |
| **19** |  | ^20^ |
| **20** |  | ^21^ |
| **21** |  | ^22–25^ |
| **22** | Critical intermediate in biosythiesis of compounds (**16**) and (**18**). | ^26–28^ |
| **23** | Possible metabolite of an industrial chemical that accumulated in insect larvae. | ^29^ |
| **24** | Metabolite of fluoroacetic acid. | ^30^ |
| **25** | Unique inorganic natural product with a Si-F bond. | ^31^ |
| **26** | Possible metabolite of an industrial chemical that accumulated in insect larvae. | ^29^ |
| **27** | The natural product status of compounds (**27**-**31**) remains to be confirmed ^15,17^. | ^32^ |
| **28** | The natural product status of compounds (**27**-**31**) remains to be confirmed ^15,17^. | ^32^ |
| **29** | The natural product status of compounds (**27**-**31**) remains to be confirmed ^15,17^. | ^32^ |
| **30** | The natural product status of compounds (**27**-**31**) remains to be confirmed ^15,17^. | ^32^ |
| **31** | The natural product status of compounds (**27**-**31**) remains to be confirmed ^15,17^. | ^32^ |

**Supplementary Appendix B:** List of structures of known natural N-halogenated compounds.

| **Compound name** | **Reference** |
| --- | --- |
| pseudoceratonic acid | ^33^ |
| N-bromo-taurine | ^34^ |
| N,N-dibromo-taurine | ^34^ |
| N-chloro-taurine | ^35^ |
| N-bromo-rhapallin A | ^36^ |
| 1,2,5-tribromo-3-bromoamino-7-bromomethylnaphthalene | ^37^ |
| 2,5,8-tribromo-3-bromoamino-7-bromomethylnaphthalene | ^37^ |
| 2,5,6-tribromo-3-bromoamino-7-bromomethylnaphthalene | ^37^ |

**Supplementary References:**

1. Ward, P. F. V, Hall, R. J. & Peters, R. A. Fluoro-fatty acids in the seeds of Dichapetalum toxicarium. *Nature* **201**, 611–612 (1964).

2. Hamilton, J. T. G. & Harper, D. B. Fluoro fatty acids in seed oil of Dichapetalum toxicarium. *Phytochemistry* **44**, 1129–1132 (1997).

3. Christie, W. W., Hamilton, J. T. G. & Harper, D. B. Mass spectrometry of fluorinated fatty acids in the seed oil of Dichapetalum toxicarium. *Chem. Phys. Lipids* **97**, 41–47 (1998).

4. Peters, R. A., Hall, R. J., Ward, P. F. V & Sheppard, N. The chemical nature of the toxic compounds containing fluorine in the seeds of Dichapetalum toxicarium. *Biochem. J.* **77**, 17 (1960).

5. Harper, D. B., Hamilton, J. T. G. & O’Hagan, D. Identification of threo-ig-fluoro. 9, 10-Dihydroxystearic acid: a novel cofluorinated fatty acid from dichapetalum toxicarium seeds. *Tetrahedron Lett.* **31**, 7661–7662 (1990).

6. Soiefer, A. I. & Kostyniak, P. J. Purification of a fluoroacetate-specific defluorinase from mouse liver cytosol. *J. Biol. Chem.* **259**, 10787–10792 (1984).

7. Seong, H. J., Kwon, S. W., Seo, D.-C., Kim, J.-H. & Jang, Y.-S. Enzymatic defluorination of fluorinated compounds. *Appl. Biol. Chem.* **62**, 1–8 (2019).

8. Moss, S. J. *et al.* Fluoroacetaldehyde: a precursor of both fluoroacetate and 4-fluorothreonine in Streptomyces cattleya. *Chem. Commun.* 2281–2282 (2000).

9. Schaffrath, C., Cobb, S. L. & O’Hagan, D. Cell‐free biosynthesis of fluoroacetate and 4‐fluorothreonine in Streptomyces cattleya. *Angew. Chemie* **114**, 4069–4071 (2002).

10. Zhao, C. *et al.* Insights into fluorometabolite biosynthesis in Streptomyces cattleya DSM46488 through genome sequence and knockout mutants. *Bioorg. Chem.* **44**, 1–7 (2012).

11. McEwan, T. Isolation and identification of the toxic principle of Gastrolobium grandiflorum. *Nature* **201**, 827 (1964).

12. Baron, M. L., Bothroyd, C. M., Rogers, G. I., Staffa, A. & Rae, I. D. Detection and measurement of fluoroacetate in plant extracts by 19FNMR. *Phytochemistry* **26**, 2293–2295 (1987).

13. Hamilton, J. G., Murphy, C. D., Amin, M. R. & Harper, D. B. Exploring the biosynthetic origin of fluoroacetate and 4-fluorothreonine in Streptomyces cattleya. *J. Chem. Soc. Perkin Trans. 1* 759–768 (1998).

14. Huang, S. Fluoroacetate biosynthesis from the marine-derived bacterium Streptomyces xinghaiensis NRRL B-24674. *Org. Biomol. Chem.* **12**, 4828–4831 (2014).

15. Deng, H., O’Hagan, D. & Schaffrath, C. Fluorometabolite biosynthesis and the fluorinase from Streptomyces cattleya. *Nat. Prod. Rep.* **21**, 773–784 (2004).

16. Peters, R. A. & Shorthouse, M. Identification of a volatile constituent formed by homogenates of Acacia georginae exposed to fluoride. *Nature* **231**, 123–124 (1971).

17. Chan, K. K. J. & O’Hagan, D. *The rare fluorinated natural products and biotechnological prospects for fluorine enzymology*. *Methods in Enzymology* vol. 516 (Elsevier Inc., 2012).

18. Carvalho, M. F. & Oliveira, R. S. Natural production of fluorinated compounds and biotechnological prospects of the fluorinase enzyme. *Crit. Rev. Biotechnol.* **37**, 880–897 (2017).

19. Murphy, C. D., O’Hagan, D. & Schaffrath, C. Identification of A PLP-Dependent Threonine Transaldolase: A Novel Enzyme Involved in 4-Fluorothreonine Biosynthesis in Streptomyces Cattleya This Work Was Supported by the Biotechnological and Biological Sciences Research Council and the University of St Andrews. *Angew. Chem. Int. Ed. Engl.* **40**, 4479–4481 (2001).

20. Ma, L. *et al.* Identification of a fluorometabolite from Streptomyces sp. MA37:(2 R 3 S 4 S)-5-fluoro-2, 3, 4-trihydroxypentanoic acid. *Chem. Sci.* **6**, 1414–1419 (2015).

21. Peters, R. A. & Shorthouse, M. Fluorocitrate in plants and food stuffs. *Phytochemistry* **11**, 1337–1338 (1972).

22. Pasternak, A. R. O., Bechthold, A. & Zechel, D. L. Identification of genes essential for sulfamate and fluorine incorporation during nucleocidin biosynthesis. *ChemBioChem* **23**, e202200140 (2022).

23. Chen, Y., Zhang, Q., Feng, X., Wojnowska, M. & O’Hagan, D. Streptomyces aureorectus DSM 41692 and Streptomyces virens DSM 41465 are producers of the antibiotic nucleocidin and 4′-fluoroadenosine is identified as a co-product. *Org. Biomol. Chem.* **19**, 10081–10084 (2021).

24. Wojnowska, M., Feng, X., Chen, Y., Deng, H. & O’Hagan, D. Identification of genes essential for fluorination and sulfamylation within the nucleocidin gene clusters of Streptomyces calvus and Streptomyces virens. *ChemBioChem* **24**, e202200684 (2023).

25. Thomas, S. O. *et al.* Nucleocidin, a new antibiotic with activity against Trypanosomes. *Nucleocidin, a new Antibiot. with Act. against Trypanos.* (1957).

26. Dong, C. *et al.* Crystallization and X-ray diffraction of 5′-fluoro-5′-deoxyadenosine synthase, a fluorination enzyme from Streptomyces cattleya. *Acta Crystallogr. Sect. D Biol. Crystallogr.* **59**, 2292–2293 (2003).

27. Schaffrath, C., Deng, H. & O’Hagan, D. Isolation and characterisation of 5′-fluorodeoxyadenosine synthase, a fluorination enzyme from Streptomyces cattleya. *FEBS Lett.* **547**, 111–114 (2003).

28. Zhu, X., Robinson, D. A., McEwan, A. R., O’Hagan, D. & Naismith, J. H. Mechanism of Enzymatic Fluorination in Streptomyces c attleya. *J. Am. Chem. Soc.* **129**, 14597–14604 (2007).

29. Gao, J., Li, W., Niu, L., Cao, R. & Yin, W. Isolation and structural elucidation of novel antimicrobial compounds from maggots of Chrysomyis megacephala Fabricius. *Nat. Prod. Res.* **29**, 239–246 (2015).

30. Aldous, J. G. The nature of the metabolites of fluoroacetic acid in bakers’ yeast. *Biochem. Pharmacol.* **12**, 627–632 (1963).

31. Gregson, R. P. *et al.* Fluorine is a major constituent of the marine sponge Halichondria moorei. *Science (80-. ).* **206**, 1108–1109 (1979).

32. Xu, X.-H. *et al.* 5-Fluorouracil derivatives from the sponge Phakellia fusca. *J. Nat. Prod.* **66**, 285–288 (2003).

33. Shaala, L. A. & Youssef, D. T. A. Pseudoceratonic acid and moloka’iamine derivatives from the Red Sea Verongiid sponge Pseudoceratina arabica. *Mar. Drugs* **18**, 525 (2020).

34. Thomas, E. L., Bozeman, P. M., Jefferson, M. M. & King, C. C. Oxidation of Bromide by the Human Leukocyte Enzymes Myeloperoxidase and Eosinophil Peroxidase: Formation of bromoamines. *J. Biol. Chem.* **270**, 2906–2913 (1995).

35. Weiss, S. J., Klein, R., Slivka, A. & Wei, M. Chlorination of taurine by human neutrophils: evidence for hypochlorous acid generation. *J. Clin. Invest.* **70**, 598–607 (1982).

36. Cen, Y. Z., Su, J. Y. & Zeng, L. M. Studies on the chemical compositions of the marine sponge Rhaphisia pallida. 1. The structural elucidation of two novel ten-membered heterocyclic compounds. *Chem. J. Chinese Univ.* **18**, 1057–1060 (1997).

37. Qin, J. *et al.* Highly brominated metabolites from marine red alga Laurencia similis inhibit protein tyrosine phosphatase 1B. *Bioorg. Med. Chem. Lett.* **20**, 7152–7154 (2010).
